# De novo and cell line models of human mammary cell transformation reveal an essential role for Yb-1 in multiple stages of human breast cancer

**DOI:** 10.1038/s41418-021-00836-6

**Published:** 2021-07-22

**Authors:** Sylvain Lefort, Amal El-Naggar, Susanna Tan, Shane Colborne, Gian Luca Negri, Davide Pellacani, Martin Hirst, Barry Gusterson, Gregg B. Morin, Poul H. Sorensen, Connie J. Eaves

**Affiliations:** 1 Terry Fox Laboratory, British Columbia Cancer Agency, Vancouver, BC Canada; 2 Department of Molecular Oncology, British Columbia Cancer Agency, Vancouver, BC Canada; 3 Faculty of Medicine, Department of Pathology, Menoufia University, Al Minufya, Egypt; 4 Department of Medicine, University of British Columbia, Vancouver, BC Canada; 5 Canada’s Michael Smith Genome Sciences Centre, British Columbia Cancer Agency, Vancouver, BC Canada; 6 Michael Smith Laboratories, University of British Columbia, Vancouver, BC Canada; 7 Department of Microbiology and Immunology, University of British Columbia, Vancouver, BC Canada; 8 Institute of Cancer Sciences, College of Medical, Veterinary and Life Sciences, University of Glasgow, Glasgow, UK; 9 Department of Medical Genetics, University of British Columbia, Vancouver, BC Canada; 10 Department of Pathology and Laboratory Medicine, University of British Columbia, Vancouver, BC Canada

**Keywords:** Cancer models, Metastasis, Oncogenes

## Abstract

Breast cancer heterogeneity has made it challenging to identify mechanisms critical to the initial stages of their genesis in vivo. Here, we sought to interrogate the role of YB-1 in newly arising human breast cancers as well as in established cell lines. In a first series of experiments, we found that short-hairpin RNA-mediated knockdown of YB-1 in MDA-MB-231 cells blocked both their local tumour-forming and lung-colonising activity in immunodeficient mice. Conversely, upregulated expression of YB-1 enhanced the poor in vivo tumorigenicity of T47D cells. We then found that YB-1 knockdown also inhibits the initial generation in mice of invasive ductal carcinomas and ductal carcinomas in situ from freshly isolated human mammary cells transduced, respectively, with *KRAS*^*G12D*^ or myristoylated-AKT1. Interestingly, increased expression of HIF1α and G3BP1, two YB-1 translational targets and elements of a stress-adaptive programme, mirrored the levels of YB-1 in both transformed primary and established MDA-MB-231 breast cancer cells.

## Introduction

Mammalian Y-box binding protein-1 (YB-1) is a member of the family of DNA/RNA binding proteins with a conserved cold-shock domain (CSD). Mammalian CSD proteins are widely expressed and involved in many fundamental processes. These include DNA repair, mRNA transcription, splicing, stabilisation and mRNA translation [1, 2]. In many tumour types, most notably those with metastatic activity, elevated YB-1 expression correlates with drug resistance and poor survival [3, 4]. In human sarcoma cells, we previously showed that YB-1 can drive metastasis through its ability to bind directly to the 5′ untranslated region (5′ UTR) of *HIF1A* [5] and *NFE2L2* [6] mRNAs and thereby enhance their translation in cells responding to diverse stress conditions. YB-1 has also been shown to promote stress-induced stress granule (SG) formation in pancreatic and colon cells [7], and we have demonstrated that, in multiple human tumour cell types, this involves the translational activation of *G3BP1* mRNAs [8]. We also previously reported that YB-1 can contribute to the acquisition of an epithelial-to-mesenchymal transition and a stress-related increase in invasive and metastatic properties of human malignant cells [9]. However, if and how YB-1 might play a role in the initial stages of malignant transformation of human cells has remained unknown, both because of the lack of patients’ samples of viable tissue at this stage and of in vivo models of de novo cancer development from cells isolated directly from normal human tissue.

To overcome this constraint, we have taken advantage of a system we have described recently that allows invasive ductal carcinomas (IDCs) to be reproducibly and rapidly obtained from freshly isolated normal human mammary cells transduced with a lentiviral vector encoding a *KRAS*^*G12D*^ cDNA [10, 11]. This approach efficiently transforms cells from two of the three major subsets of human mammary cells, specifically the two that proliferate in response to epidermal growth factor (EGF). These two cell types are referred to as basal cells (BCs) and luminal progenitors (LPs), the latter representing a phenotypically and biologically distinct subset of the luminal cell compartment [12]. Tumours produced when several thousand *KRAS*^*G12D*^–transduced BCs or LPs are transplanted into immunodeficient mice are highly polyclonal, histologically classified as invasive ductal carcinomas (IDCs) but phenotypically heterogeneous, with a variable content of cells positive for oestrogen receptor (ER), progesterone receptor (PR), human epidermal growth factor receptor-2 (HER2), EGFR, Ki67 and cytokeratins (CK) 8/18 [10].

Here we demonstrate a shared in vivo growth dependence on YB-1 of transformed human mammary cells that span a broad spectrum of tumorigenic activity in transplanted immunodeficient mice. This includes not only two human breast cancer cell lines with different in vivo tumour-initiating and metastatic activities. It also includes the IDCs generated de novo from cells of *KRAS*^*G12D*^-induced BCs and LPs, as well as a new model of ductal carcinoma in situ (DCIS) that we now find can be produced de novo from normal human mammary BCs or LPs transduced with a vector encoding a myristoylated form of *AKT1* (*myr-AKT*). We also show that the level of YB-1 activated correlates with acquisition of known features of a cytoprotective stress response [8, 9].

## Materials and methods

### Cells and cultures

Normal human reduction mammoplasty discard tissue was collected with informed consent, according to protocols approved by the University of British Columbia Research Ethics Board (#H19-03794). Tissue fragments were then isolated and viably cryopreserved [13]. As required, thawed fragments were rinsed with 2% fetal bovine serum (FBS, from STEMCELL Technologies) in Hank’s Balanced Salt Solution (HF), and the cells then dissociated in 2.5 mg/ml trypsin with 1 mM EDTA and 5 mg/ml dispase (STEMCELL Technologies) with 100 μg/ml DNaseI (Sigma) and washing of the cells with HF between each step. The resulting cell suspension was filtered through a 40 μm mesh and BCs then isolated by FACS according to their CD45^−^CD31^−^EpCAM^lo^CD49f^+^ phenotype, LPs according to their CD45^−^CD31^−^EpCAM^hi^CD49f^+^ phenotype, luminal cells (LCs) according to their CD45^−^CD31^−^EpCAM^hi^CD49f^−^ phenotype and stromal cells (SCs) according to their CD45^−^CD31^−^EpCAM^−^CD49f^−^ phenotype using well established protocols and reagents [14]. Following FACS, cells were transduced or cultured in SF7 media supplemented with 5% FBS. MCF10A cells (obtained from J Brugge, Harvard University, Cambridge, MA) were maintained in phenol-free DMEM/F12 nutrient mix supplemented with 5% horse serum, 10 mg/ml insulin, 0.5 mg/ml hydrocortisone, 100 ng/ml cholera toxin, 20 ng/ml EGF (all from Sigma) and 1% penicillin/streptomycin (Life Technologies). 3D assays of human mammary cells were performed by culturing the cells in the presence of irradiated 3T3 fibroblasts for 8, 10 or 14 days in Matrigel (Corning) SF7 media supplemented with 5% FBS as previously described [15]. MDA-MB-231 cells were originally obtained from ATCC and maintained in DMEM with 10% FBS. Their identity was confirmed by DNA sequencing, including detection of the *KRAS*^*G13D*^ allele [16]. T47D cells were obtained from Joanne Emerman (Department of Anatomy, University of British Columbia) and maintained in RPMI with 10% FBS. For 3D cultures, cells were transferred to ultra-low attachment surface plates and cultured for 24 h before harvesting. Primary cells were transduced with lentiviral vectors as previously described [10].

### Vectors

MND vectors encoding *KRAS*^*G12D*^, *luciferase* or *myrAKT* cDNAs were constructed and produced in U293 cells as previously described [10]. For stable inhibition of YBX1 transcripts, lentiviral vectors encoding sh*YBX1* (sc-38634-V, Santa Cruz, #1307/TRCN0000315307, #1309/TRCN0000315309, and #948/TRCN0000007948, Sigma), or sh*Scr* (sc-108080, Santa Cruz or shScr (SHC202, Sigma) were purchased or constructed. Inducible *KRAS*^*G12D*^- and *YB-1*- encoding vectors were generated in a pINDUCER21 backbone [17] by replacing the attR1-ORF-attR2 cassette with a *KRAS*^*G12D*^−2A-*Kusabira Orange* (KO) cassette. Thus, cells transduced with this vector express GFP constitutive and, in the presence of doxycycline (Dox), co-expression of *KRAS*^*G12D*^ and KO is induced. *KRAS*^*G12D*^ expressing Dox-treated cells could thus be quantified by flow cytometric detection of the KO that served as a reporter for KRAS expression, as well as by direct RT-PCR of *KRAS*^*G12D*^ transcripts.

### Xenografts

Female immunodeficient NOD-*Rag1*^*−/−*^*IL2Rγc*^*−/−*^ (NRG) mice were bred and housed in the animal facility at the British Columbia Cancer Research Centre under SPF conditions. Injections were performed on 7- to 10-week-old mice. All experimental procedures were approved by the University of British Columbia Animal Care Committee. Mice were xenografted with cells containing inducible vectors were supplied with Dox (1 mg/ml) in their drinking water as indicated.

To generate primary tumours, enzymatically dissociated human mammary cell suspensions were prepared, transduced and transplanted into mice subcutaneously (SQ) in 50% (v/v) Matrigel [10]. To measure tumour bioluminescence from a co-transduced luciferase cDNA, mice were injected intraperitoneally with 150 mg/kg body weight of d-luciferin (Promega) and 10 min later the mice were imaged using a Xenogen IVIS Lumina system with Living Image version 3.0 software (Caliper Life Sciences). To prepare single cell suspensions from excised tumours, the tissue was minced with a scalpel, incubated at 37 °C in DMEM/F12 media supplemented with 5% FBS and 300 U/ml collagenase and 100 U/ml hyaluronidase for 1–2 h with periodic vortexing, washed with HF and treated with 2.5 mg/ml trypsin with 1 mM EDTA and 5 mg/ml dispase with 100 μg/ml DNaseI. Human cells were sorted after staining with anti-human specific antibodies directed against EpCAM and CD298 (Biolegend) with simultaneous depletion of mouse cells stained with anti-mouse-specific antibodies directed against CD45 and CD31 (Biolegend).

### Immunohistochemical analyses

Pieces of tumours obtained from mice or normal breast were fixed in 10% buffered formalin (Fisher), washed in 70% ethanol, processed and embedded in paraffin. Sections of paraffin-embedded tissue (3 mm) were first treated with Target Retrieval solution (DAKO) and then a cytomation serum-free protein block (DAKO) followed by staining with specific antibodies recognising human YB-1 (#HPA040304, Sigma), ER (SP1; 1/50; Thermofisher; RM9101), PR (SP2; 1/50; Neomarker; 9102), Ki67 (SP6; 1/50; Thermofisher; RM9106), CK14 (Novocastra/Leica; 1/50; NCL-L-LL02), CK8/18 (Novocastra/Leica; 1/50; NCL-L-5D3), p63 (4A4; 1/50; Gentex; GTX23239), SMA (1A4; 1/100; Dako; MO851), HIF1a (Novus Biologicals; 1/150; NB100-134), CAIX (R&D Systems; 1/200; AF2188), CD34 (Abcam; 1/400; Ab81289), NRF2 (Abcam. 1/150; Ab62352) and G3BP (Novus Biologicals; 1/500; NBP2-16563). A secondary mouse or rabbit antibody conjugated to horseradish peroxidase and treatment with 3,3′-diaminobenzidine (DAB, DAKO) was used to obtain a positive brown staining. Negative IgG controls were performed on normal reduction mammoplasty tissue.

Quantitative immunohistochemical (IHC) analysis of stained samples was conducted using the colour deconvolution plugin which implements stain separation and the ImmunoRatio plugin for ImageJ software (developed at the National Institutes of Health, USA, and available at http://rsb.info.nih.gov/ij/). Student’s *t* test was used for data analysis, unless indicated otherwise.

### Western blot analyses

After the required treatment, cells were washed with cold PBS and incubated for 15 min at 4 °C with RIPA lysis buffer (30 mM Tris-HCl, pH 7.5, 150 mM NaCl, 10% glycerol, 1% Triton X-100 (Sigma) supplemented with a 1 mM NaF, 1 mM NaVO3 and 1 mM PMSF (all Sigma). Cells extracts were centrifuged at 13,000×*g* for 10 min at 4 °C. The protein concentration of the supernatant fraction was determined using the Bio-Rad Bradford Protein Assay Kit according to the manufacturer’s instructions. For each sample, an equal amount of total protein was diluted in sample buffer (Invitrogen) and boiled for 5 min. Samples were loaded onto precast NuPAGE 4–12% polyacrylamide gels (Invitrogen). After electrophoresis, proteins were transferred to a PVDF transfer membrane. Membranes were then blotted overnight at 4 °C with appropriate primary antibodies, such as anti-ACTIN (Santa Cruz, sc-1615, 1/10,000), anti-H3 (Cell Signaling Technology, 12648, 1/10,000), anti-RAS (Cell Signaling Technologies, 3339, 1/1,000), anti-YB-1 (Cell Signaling Technology, 4202, 1/1,000) and anti-HIF1α (Cayman; 10006421; 1/1000). Specific binding of antibodies was detected using appropriate secondary antibodies conjugated to horseradish peroxidase, and visualised with SuperSignal™ West Femto Maximum Sensitivity Substrate (Thermofisher) on a ChemiDoc Gel Imaging system (Bio-rad). Densitometric analyses of immunoblots were performed using ImageJ.

### RNAseq data

Copy number alterations and Z-score normalised RNAseq expression values (V2 RSEM) were obtained from cBioPortal [18], from TCGA [19], METABRIC [20] and mutational profiles of metastatic breast cancers [21] datasets. Paired-end reads were generated from MDA-MB-231 shScr or shYBX1 cells or derived tumours, on an Illumina HiSeq2500 sequencer. Read sequences were aligned to the hg19 human reference using the BWA-SW algorithm [22] to generate binary alignment/map (BAM) files. Transcript counts were obtained with the summarise Overlaps function from GenomicAlignments package [23]. Differential expression analysis was performed with DESeq2 package [24].

### Proteomic data

Tissues were thawed and lysed in 100 μL lysis buffer containing 500 mM Tris-HCL pH 8, 2% SDS (w/v), 1% NP-40 (v/v), 1% Triton X100 (v/v), 0.5 mM EDTA, 50 mM NaCl, 10 mM Tri(2-carboxyethyl)phosphine (TCEP) and 40 mM chloroacetamide (CAA). The proteins were then denatured by heating at 95 °C for 90 min with shaking at 1,100 rpm before incubation at room temperature for 90 min in the dark to allow reduction and alkylation of disulfide bonds by TCEP and CAA respectively. SP3 beads [25, 26] were added and the tissues were sonicated in a Bioruptor Pico (Diagenode) for 10 cycles (30 s ON, 30 s OFF). The samples were purified and prepared for trypsin digestion using the SP3 method [26]. Tryptic peptides from each sample were individually labelled with TMT 10-plex labels (Thermo Scientific), pooled, and fractionated into 12 fractions by high pH RP-HPLC, desalted, and then analysed using an Easy-nLC1000 liquid chromatograph (LC) (Thermo Scientific) coupled to a Orbitrap Fusion Tribrid mass spectrometry (MS) (Thermo Scientific) operating in MS3 mode. The offline peptide fractionation and LC-MS conditions are as described [26]. The raw MS data were searched using Proteome Discoverer (version 2.1.1.21) using the embedded Sequest HT algorithm against a combined UniProt Human proteome database with a list of common contaminants appended (24,624 total sequences). Sequest HT parameters were specified as: trypsin enzyme, allowance for 2 missed cleavages, minimum peptide length of 6, precursor mass tolerance of 20 ppm, and a fragment mass tolerance of 0.6. Dynamic modifications allowed were oxidation of methionine residues, and TMT at lysine residues and peptide N-termini. Carbamidomethylation of cysteine residues was set as a static modification. Peptide spectral match (PSM) error rates were determined using the target-decoy strategy coupled to Percolator modelling of positive and false matches [27, 28]. Data were filtered at the PSM-level to control for false discoveries using a *q*-value cutoff of 0.05 as determined by Percolator. Contaminant and decoy proteins were removed from all datasets prior to downstream analysis. Statistical analysis of differential protein expression was performed at the peptide level using a modified version of the PECA function that is appropriate for input of log-transformed data [29]. PECA uses Limma [30] to generate a linear model for estimating fold changes and standard errors prior to empirical Bayes smoothing. Median t-statistics of the assigned peptides were used to calculate false-discovery rate-adjusted *p*-values determined from the beta distribution, as described previously [29].

### RT-PCR

Total RNA was extracted from cryopreserved tumour samples or cultured cells using the Total RNA Isolation Micro kit (Agilent) and cDNA then synthesised using SuperScript VILO cDNA synthesis kit (Life Technologies). RT-PCR was performed using a SYBR Green master mix (Applied Biosystems) and samples run in triplicate with custom-designed primers. KRAS expression in tumours was confirmed using primers for KRAS (5′-ACA CAA AAC AGG CTC AGG ACT-3′; 5′-AGG CAT CAT CAA CAC CCT GT-3′) and GAPDH (5′-ACG TAC TCA GCG CCA GCA TC-3′; 5′-ACC GTC AAG GCT GAG AAC GG-3′) as normaliser control.

### Statistical analyses

Values are expressed as mean ± SEM, unless otherwise specified. Significance was evaluated using Student’s *t* test, unless otherwise specified and indicated as follows: *P < 0.05, **P < 0.01, ***P < 0.001, ns not significant.

## Results

### Altered KRAS and AKT activity is associated with increased YB-1 expression and indicators of activated stress responses in patients’ breast cancers

**Fig. 1:**
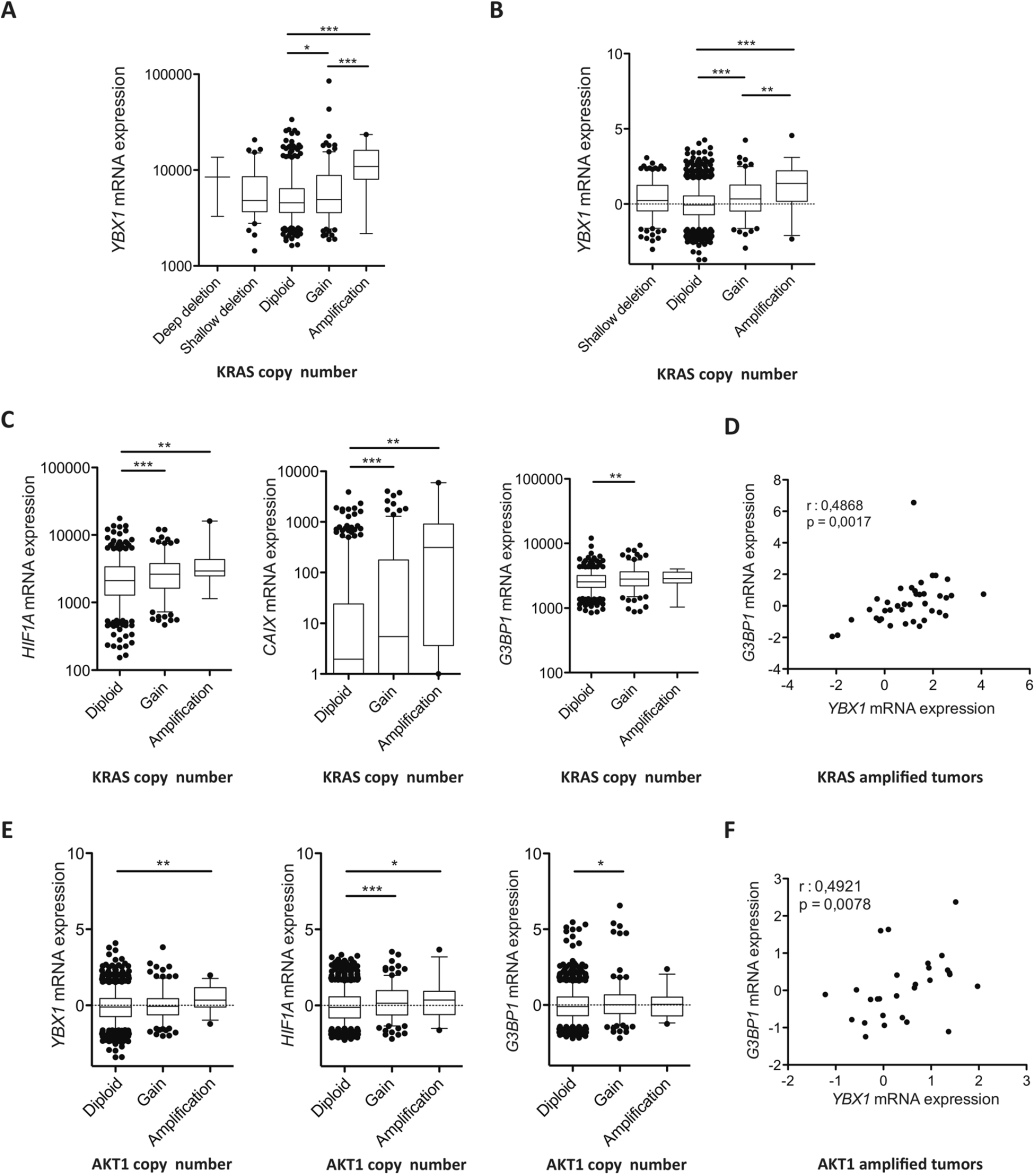
Patients’ *KRAS*-amplified tumours express high levels of *YB-1* and associated stress response genes. **A** *YB-1* mRNA levels compared to *KRAS* copy number status in invasive breast carcinoma samples in the TCGA dataset. Values for *YB-1* are shown as Reads Per Kilobase of transcript, per Million mapped reads (RPKMs). **B** *YB-1* mRNA levels compared to *KRAS* copy number status in invasive breast carcinoma samples in the METABRIC dataset. Values for *YB-1* are shown as RPKMs. **C** *HIF1A* (left panel) *CAIX* (middle panel) and *G3BP1* (right panel) mRNA expression according to *KRAS* copy number status in invasive breast carcinomas in TCGA dataset. Values for *HIF1A, CAIX and G3BP1* are shown as RPKMs. **D** Scatter plot of *YB-1* and *G3BP1* mRNA expression in amplified-*KRAS* invasive breast carcinomas. **E** *YB-1* (left panel) *HIF1A* (middle panel) and *G3BP1* (right panel) mRNA expression according to *AKT1* copy number status in invasive breast carcinomas in METABRIC dataset. Values for *YB-1*, *HIF1A and CAIX* are shown as RPKMs. **F** Scatter plot of *YBX1* and *G3BP1* mRNA expression in amplified-*AKT1* invasive breast carcinomas. *P*-values in panels (**A**, **C**, **D**) were determined by Student’s *t* test, *P < 0.05, **P < 0.01, ***P < 0.001.

Examination of three large breast cancer datasets show that elevated levels of *YBX1* (hereafter referred to as *YB-1*) transcripts are associated with a reduced overall survival of patients with ER- breast cancers, most notably in those with metastatic disease [19–21]. Elevated expression of YB-1 is also positively associated with a gain of function or amplification of the *KRAS, ERBB2* and *PIK3CA* genes, or deletions of the *TP53* gene, but not with amplified *HRAS* or *NRAS* mutations (Fig. 1A, B, and Appendix Fig. S1A–D and data not shown). Previous studies have indicated that YB-1 can alter the translational control in malignant cells of a large number of proteins involved in cytoprotective responses to stresses they encounter, including HIF1α, G3BP1 and NRF2 [5, 6, 8, 31]. From analyses of the same three patient breast cancer datasets, we find *HIF1A* transcripts and its transcriptional targets, *CAIX* and *VEGFA* [32], and *G3BP1* (but not *NRF2*) are all significantly higher in breast cancers that exhibit gain of function or amplified *KRAS* (Fig. 1C, D and Appendix Fig. S2A–C). Elevated levels of *HIF1A*, its target *CAIX*, and *VEGFA* transcripts are also seen in tumours with a gain of function or amplification of *YB-1*, although this was not the case for *G3BP1* or *NFE2L2* (encoding NRF2; Appendix Fig. S2D). Analyses of these datasets further reveals *YB-1, HIF1A, CAIX* and *G3BP1* transcript levels to be increased in tumours with amplified *AKT1* and/or increased *AKT1* expression (Fig. 1E, F and Appendix Fig. S2E–H). Mutations in *AKT1* are of interest as these alterations and the pathways they affect are prevalent but differ in DCIS versus IDC [33–35]. This suggested that YB-1 may also play a role in pre-neoplastic changes in mammary cells as well as in populations with more advanced features of malignancy.

### YB-1 is required for the in vivo tumorigenic and metastatic activities of MDA-MB-231 cells

**Fig. 2:**
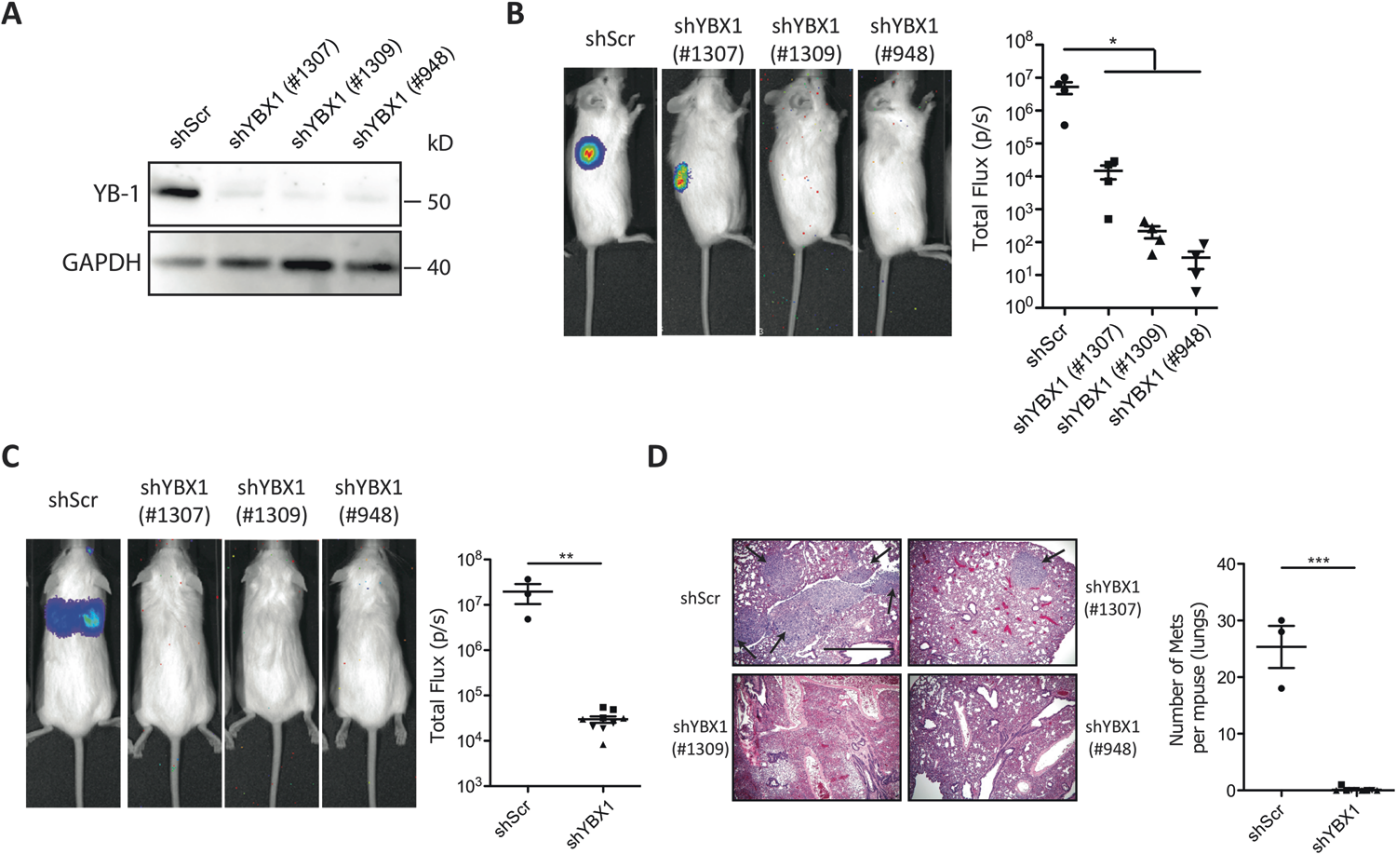
Suppressed YB-1 expression impairs the generation of local and metastatic tumour formation by the MDA-MB-231 cells. **A** Western blots of MDA-MB-231 cells transduced with *shYB-1(#1307), shYB-1(#1309)* or *sh*Y*B-1(#948)* or a control *Scr* vector, showing YB-1 protein levels (relative to GAPDH). **B** Representative photographs of bioluminescence signals in mice injected SQ with MDA-MB-231 cells transduced with *shYB-1(#1307), shYB-1(#1309), shYB-1(#948)* or *shScr*. Dot plots show the bioluminescence signals exhibited by these tumours 34 days post-transplant. Data are from individual mice (*n* = 4). **C** Representative pictures of the bioluminescence signals obtained in mice 34 days after being injected intravenously with MDA-MB-231 cells transduced with *shYB-1* or *shScr* vectors. The dot plot shows the measured bioluminescence signals exhibited by the progeny of these cells generated in vivo. Data are from individual mice (shScr *n* = 3; shYBX1 *n* = 9). **D** Representative photomicrographs of H&E- or YB-1-stained sections of lungs of mice injected intravenously with MDA-MB-231 cells transduced with *shYB-1* or *shScr* vectors. The dot plot shows the number of metastases per mouse (both lungs). Data are from individual mice (shScr *n* = 3; shYBX1 *n* = 9). *P*-values in panels **B**–**D** were determined by Student’s *t* test, *P < 0.05, **P < 0.01, ***P < 0.001.

As a first approach to examining the biological significance of elevated YB-1 expression on the in vivo tumorigenic properties of human breast cancer cells with altered *KRAS* or *AKT* expression, we focused on determining the effect of reducing YB-1 protein levels in MDA-MB-231 cells. This human breast cancer cell line contains a *KRAS*^*G13D*^ mutation [16], displays elevated YB-1 expression [36] and displays aggressive tumorigenic and metastatic activities when transplanted into highly immunodeficient female mice [11, 37]. Transduction of these cells with lentiviral vectors encoding short hairpin (*sh*)*YB-1* constructs reduced their YB-1 protein content by 90% compared to controls (Fig. 2A and Appendix Fig. S3A–B). Reduced YB-1 expression, in turn, caused a marked decrease in both the size (Fig. 2B and Appendix Fig. S3C, D) and YB-1 content (Appendix Fig. S3E) of the tumours produced from the transduced cells at their SQ sites of injection. Parallel experiments in which mice were injected intravenously with the same test or control shRNA-transduced cells showed dramatically reduced metastatic activity of the *shYB-1*-transduced cells in the lungs as compared to control cells (Fig. 2C, D and Appendix Fig. S3F–H). Taken together, these results demonstrate the strong YB-1 dependence of both the in situ and metastatic growth of MDA-MB-231 cells in xenografted immunodeficient mice.

### Loss of MDA-MB-231 cell tumorigenic activity by YB-1 inactivation is associated with evidence of a suppressed stress response

**Fig. 3:**
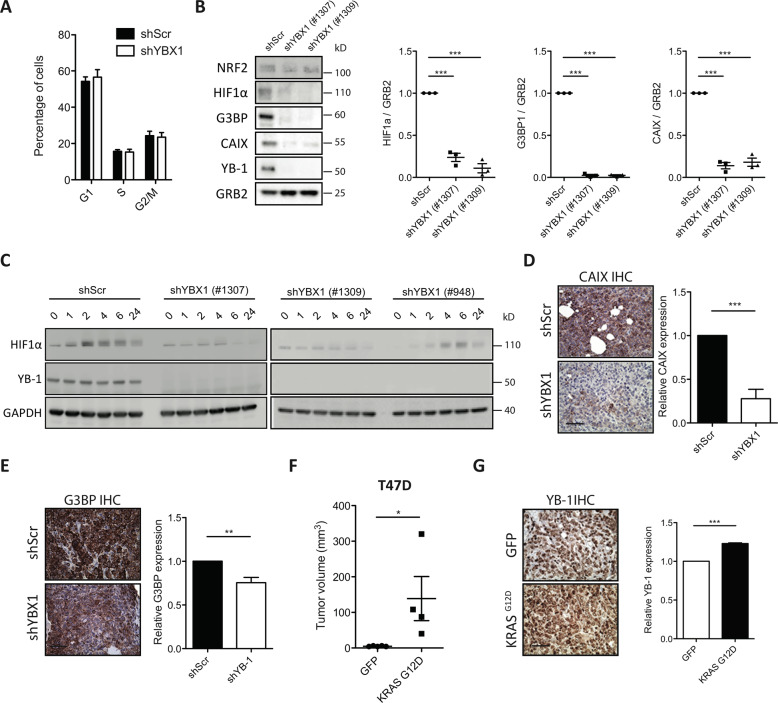
Loss of tumorigenic activity by YB-1 inactivation is associated with a suppression of stress response proteins. **A** Cell cycle analysis of *shYB-1*- or *shScr*-transduced MDA-MB-231 cells. **B** Western blots showing NRF2, HIF1α, G3BP1, CAIX and YB-1 levels (relative to GRB2) of MDA-MB-231 cells transduced with *shYB-1 or shScr* vectors grown in ultra-low attachment plates for 24 h. Individual data are from independent experiments (*n* = 3). **C** Western blots showing HIF1α and YB-1 levels (relative to GAPDH) in *shYB-1* and *shScr*-transduced MDA-MB-231 cells maintained in vitro for up to 24 h in 1% O_2_. **D**, **E** Representative images of CAIX (**D**)-, G3BP (**E**)- stained sections of tumours produced from *shYB-1*- or *shScr*-transduced MDA-MB-231 cells. Scale bar, 100 μm. Bar graph shows measured levels of CAIX or G3BP (staining intensity). **F** Dot plots of tumour sizes obtained in mice injected SQ with T47D cells transduced with GFP-control and *KRAS*^*G12D*^ and assessed 30 days post-transplant. Data are from individual mice (GFP, *n* = 3; KRAS^G12D^ *n* = 9). **G** Representative views of YB-1 levels in the IHC-stained in vivo progeny of GFP-control and *KRAS*^*G12D*^-transduced T47D cells. Bar graph shows quantification of YB-1 expression. Data are from individual mice (GFP, *n* = 3; KRAS^G12D^, *n* = 9).

Despite the marked effects of reduced YB-1 levels on the growth of the *shYB-1*-transduced MDA-MB-231 cells in vivo, cell-cycle distribution analyses showed no evidence of alterations in their proliferative activity in standard 2D cultures (Fig. 3A), consistent with previous reports [5]. However, even a 24-h incubation of the *shYB-1*-transduced MDA-MB-231 cells under non-adherent culture conditions [6] reduced the expression of HIF1α, CAIX, G3BP1 and NRF2 in comparison to similarly cultured control-transduced cells (Fig. 3B). HIF1α expression was also reduced in *shYB-1*-transduced MDA-MB-231 cells cultured under hypoxic conditions in comparison to their control vector-transduced counterparts (Fig. 3C).

To investigate the possibility that these effects might also be operative during MDA-MB-231-induced tumour formation in vivo, we performed RNAseq and proteomic analyses of cells isolated from the small tumours derived from MDA-MB-231 cells transduced with *shYB-1*, and compared the results to data obtained from simultaneously harvested control tumours (Appendix Fig. S4A–D). This revealed numerous genes whose expression was altered by reducing YB-1 (Appendix Fig. S4B–D), including several related to hypoxia (e.g., *PPIF*, *SLC3A2* and *SLC7A1*) [38–40] and found to be elevated in breast cancers with increased expression or amplification of *YB-1* (Appendix Fig. S4E). Immunostaining of tumours generated from the *shYB-1*-transduced MDA-MB-231 cells also showed decreased levels of CAIX and G3BP compared to the control tumours (Fig. 3D, E).

We next asked whether the deregulated KRAS activity and associated *YB-1* dependence might be elicited in a human breast cancer cell line with less aggressive features of transformation. We therefore examined the properties of T47D cells following their transduction with our lentiviral *KRAS*^*G12D*^ vector [10]. The transduced T47D cells showed a predicted activation of the RAS/RAF/MEK pathway [41] (Appendix Fig. S5A, left panel) and, in transplanted mice, produced tumours that were larger and grew faster than controls (Fig. 3F).

IHC analysis of the tumours derived from the *KRAS*^*G12D*^-transduced cells demonstrated elevated levels of YB-1, G3BP1, HIF1α and CAIX compared to the control T47D tumours (Fig. 3G and data not shown). In addition, when the *KRAS*^*G12D*^-transduced T47D cells were cultured in non-adherent suspension cultures, they also showed an increased expression of HIF1α and YB-1 compared to controls (Appendix Fig. S5B), but not when cells were cultured under standard adherent 2D conditions (Appendix Fig. S5A, right panel).

Together, these results show that forced expression of KRAS^G12D^ promotes an enhanced level of expression of YB-1 and the consequent ability YB-1 to rapidly activate the expression of stress-ameliorating genes involved in response to hypoxia and other stresses that are required for tumour formation in vivo.

### YB-1 expression is an essential step in the *KRAS*^*G12D*^-mediated de novo transformation of normal human mammary cells

**Fig. 4:**
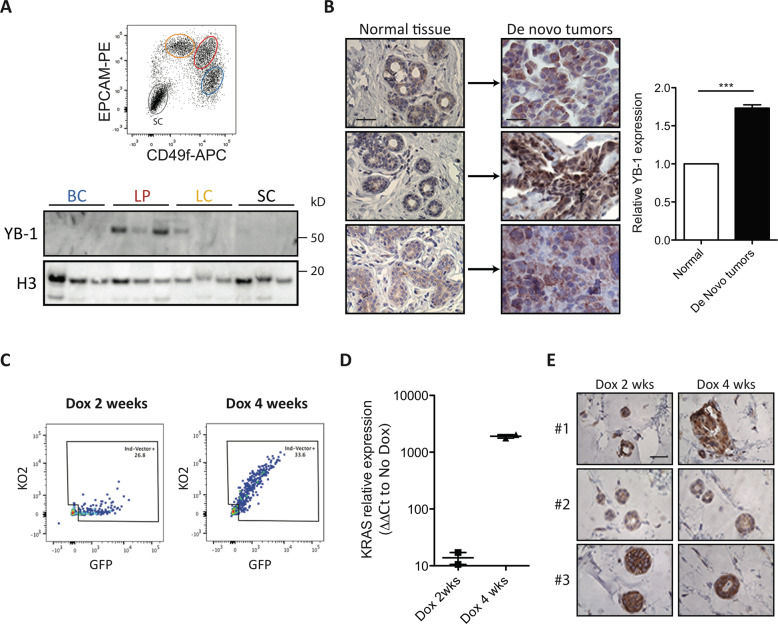
*KRAS*^*G12D*^ upregulates YB-1 in de novo KRAS^*G12D*^-transformed normal human mammary cells. **A** Western blots showing YB-1 levels (relative to H3) in human BCs, LPs, LCs and stromal cells (SCs) isolated viably from three normal donors according to their differential surface expression of EPCAM and CD49f levels and absence of expression of CD45 and CD31 (top panel). **B** Representative views of YB-1 immunostaining of normal human mammary tissue (left) and 8-week tumours derived from *KRAS*^*G12D*^-transduced normal mammary cells isolated from the same three normal donors (right). Scale bar, 50 μm. Bar graph shows quantification of YB-1 expression. Data are from individual tumours (Normal, *n* = 8; de novo tumours, *n* = 8). **C** Representative FACS profile of a 4-week xenograft of inducible *KRAS*^*G12D*^-transduced human BCs obtained from mice maintained post-transplant on doxycyline-supplemented water (Dox) for just the first 2 weeks (left panel) or for the full 4 weeks (right panel) of the experiment. **D** *KRAS* mRNA levels measured in 4-week xenografts of inducible *KRAS*^*G12D*^-transduced cells obtained from mice maintained on Dox for the first 2 weeks only, or the full 4 weeks of the experiment, as shown. The dot plot shows KRAS relative expression compared to the No Dox control mice (*n* = 2), as ΔΔCt values. **E** Representative views of YB-1 immunostaining of 4-week tumours derived from mice transplanted with cells transduced with a Dox-inducible *KRAS*^*G12D*^ cDNA and maintained on Dox for the just the first 2 weeks or the full 4 weeks before the tumours were harvested for analysis (*N* = 3 donors). Scale bar, 50 μm.

We then asked whether elevated *YB-1* expression might be similarly involved in the initial stages of human mammary tumorigenesis driven by experimentally deregulating KRAS activity in freshly isolated normal human mammary cells [10]. Initial western blot (WB) analyses of FACS-purified isolates of BCs and LPs, and also of LCs isolated directly from normal human mammary glands, showed that YB-1 protein levels are highest in normal LPs and barely detectable in BCs or LCs (Fig. 4A). Parallel analyses of *KRAS*^*G12D*^- transduced purified BCs- or LPs maintained in vitro either in standard 2D adherent cultures for 4 days (Appendix Fig. S6A) or in 3D-Matrigel cultures for up to 15 days (Appendix Fig. S6B) showed no evidence of altered YB-1 expression. However, 2 weeks after the transduced cells were injected SQ into mice (10^3^ to 2 × 10^4^ transduced cells in 100 μL of 50% Matrigel/site), the nascent tumours already evident by that time showed strong increases in YB-1 expression by IHC as compared to simultaneously stained samples of the original breast tissue samples from which the transduced cells had been isolated (Fig. 4B and Appendix Fig. S6C). In addition, the latter showed lower levels of YB-1 expression largely restricted to the cytoplasm of cells in the luminal layer (Fig. 4B and Appendix Fig. S6C). Moreover, the increased levels of YB-1 evident after just 2 weeks of growth of the *KRAS*^*G12D*^-transduced cells in vivo were already similar to those apparent in the larger tumours produced from the same cells harvested 4–6 weeks later and then stained and analysed in parallel (Fig. 4B).

To determine if sustained expression of KRAS^G12D^ is necessary to maintain the high levels of YB-1 evident within 2 weeks of growth in vivo of *KRAS*^*G12D*^-transduced primary human mammary cells, we repeated the preceding experiment but, this time, using a doxycycline (Dox)-inducible *KRAS*^*G12D*^ to transduce the cells subsequently transplanted (4–30 × 10^4^ cells injected/site). With this vector, all transduced cells express GFP, but only following exposure to Dox is co-expression of both *KRAS*^*G12D*^ and the linked indicator KO fluorochrome induced (see Methods section and Appendix Fig. S6D–F). All injected mice received Dox for the first 2 weeks to induce the initial expression of *KRAS*^*G12D*^ for that period. Then, for the next 2 weeks, half of the mice were continued on Dox, but the other half were switched back to normal drinking water (Fig. 4C). At the end of the 4-week period, transplants isolated from the mice maintained on Dox for the full 4 weeks contained predominantly KO + (*KRAS*^*G12D*^-expressing) cells (Fig. 4C; right panel) and an accompanying large (100-fold) increase in *KRAS* transcripts as compared to the tumours obtained from mice given Dox for only the first 2-weeks post-transplant (Fig. 4C, D). However, despite the loss of *KRAS* transcripts seen 2 weeks after the Dox was discontinued, these cells still showed elevated *YB-1* protein levels that were similar to those evident in the tumours obtained from cells in which *KRAS*^*G12D*^ expression had been sustained (Fig. 4E). These results suggest that in vivo, *KRAS*^*G12D*^ leads to rapidly increased *YB-1* expression in primary cells that can then persist even in the absence of continued *KRAS*^*G12D*^ expression.

### Increased AKT-drives the de novo development of DCIS from primary cells and an increase in YB-1 expression, but YB-1 alone does not confer tumorigenic activity

**Fig. 5:**
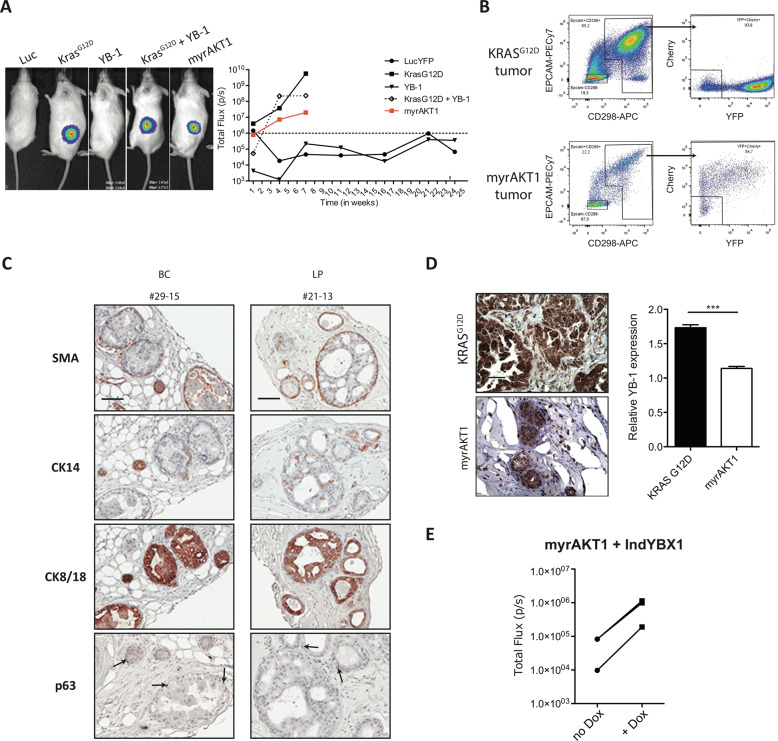
De novo formation of DCIS-like tumours leads to modestly increased levels of YB-1. **A** Representative photos of bioluminescence signals measured in mice injected SQ 7 weeks earlier with human mammary cells transduced with lentiviral vectors encoding Luc-YFP alone or in combination with *KRAS*^*G12D*^-, YB-1-, *KRAS*^*G12D*^+YB-1- or *myrAKT1*. BCs and LPs from three donors were pooled separately before transduction. Graph plot shows changes in bioluminescence activity over time. **B** Representative FACS plots of human (CD298/EPCAM)^+^ and mCherry (myrAKT1)^+^ or YFP (KRAS^G12D^)^+^ cells present in dissociated tumours generated from human mammary cells transduced with *KRAS*^*G12D*^ or *myrAKT1*. **C** Representative images of SMA-, CK14-, CK8-18- and p63-stained sections of *myrAKT1-*derived tumours initiated from either BCs or LPs. Scale bar, 100 μm. **D** Representative views of YB-1 immunostaining of 18-week primary *myrAKT1*-derived tumours generated from normal mammary cells from three different donors (#1–3). The bar graph shows a comparison of YB-1 staining intensity in *KRAS*^*G12D*^- (*n* = 10) or *myrAKT1*-derived (*n* = 6) tumours. **E** Dot plot of the bioluminescence measured in mice injected SQ with *myrAKT1*+inducible YB-1-transduced human mammary cells and given water with or without DOX. *N* = 3 donors.

We next designed experiments to determine whether increased expression of YB-1 might be important at an even earlier stage of development of human breast cancers driven by an oncogene associated with DCIS development in patients. For this, we transduced separate pools of BCs and LPs from three different normal breast tissue samples with a Luc-YFP vector for bioluminescent monitoring and a cDNA vector encoding a *myrAKT* sequence targeting the protein to the cell membrane [42] linked to mCherry. We also tested cells co-transduced with a YB-1-mCherry vector with and without the *KRAS*^*G12D*^-YFP vector, with the *KRAS*^*G12D*^-YFP vector alone as a positive control, or with the Luc-YFP vector alone as a negative control (Fig. 5A).

Bioluminescence tracking of the transplanted mice showed that ectopic expression of YB-1 alone was insufficient to confer tumour-forming capacity on primary human mammary cells and, the addition of *KRAS*^*G12D*^ expression in the same cells, did not enhance the growth of the tumours generated from cells transduced with *KRAS*^*G12D*^ alone (Fig. 5A). However, forced expression of constitutively active *myrAKT* alone led to significantly increasing luciferase signals over an 8-week period post-transplant, albeit at consistently lower levels than those obtained from transplants of *KRAS*^*G12D*^-transduced cells (Fig. 5A). Phenotypic analysis of cells generated from *myrAKT-*transduced cells showed that they were universally human EpCAM^+^CD298^+^ as well as mCherry^+^ in 9 of 13 experiments with different donors’ cells (Fig. 5B and Appendix Fig. S7A).

Histological analysis of fixed samples of the xenografted cells obtained from the recipients of the *myrAKT1*-transduced cells showed that they contained structures that closely resemble patients’ DCIS. This included a confined organisation of the cells in duct-like structures with an outer layer of cells with basal features (expression of smooth muscle actin and TP63, Fig. 5C and Appendix Fig. S7B), and extensive luminal filling by cells with luminal features (strong CK14 and CK8/18 positivity, Fig. 5C and Appendix Fig. S7B). Tumours also contained a low frequency of ER^+^ and/or Ki67^+^ cells, and PR^+^ cells were not detected (Appendix Fig. S7C). Importantly, these structures also showed an increased content of YB-1 protein as compared to cells present in normal mammary glands, but at lower levels than those typical of cells in the *KRAS*^*G12D*^-induced tumours (Fig. 5D). Notably, mice transplanted with cells co-transduced with the Dox-inducible YB-1-KO vector (Appendix Fig. S8A) plus the *myrAKT*-mCherry vector, when treated with Dox, showed increased luciferase activity (Fig. 5E), along with increased YB-1 expression, as compared to identically transplanted mice not treated with Dox (Appendix Fig. S8B).

These experiments extend the range and genetic determinants of de novo transformed primary human mammary cell types that show upregulation of YB-1 to include a DCIS model that does not involve deregulation of KRAS. At the same time, they show that a forced increase in YB-1 expression can enhance the growth of DCIS tumours in the *myrAKT* model, although increased YB-1 alone does not appear to confer any tumorigenic activity in this setting.

### *KRAS*^*G12D*^ requires YB-1 to induce its tumorigenic activity and the HIF1α response in transduced normal human mammary cells

**Fig. 6:**
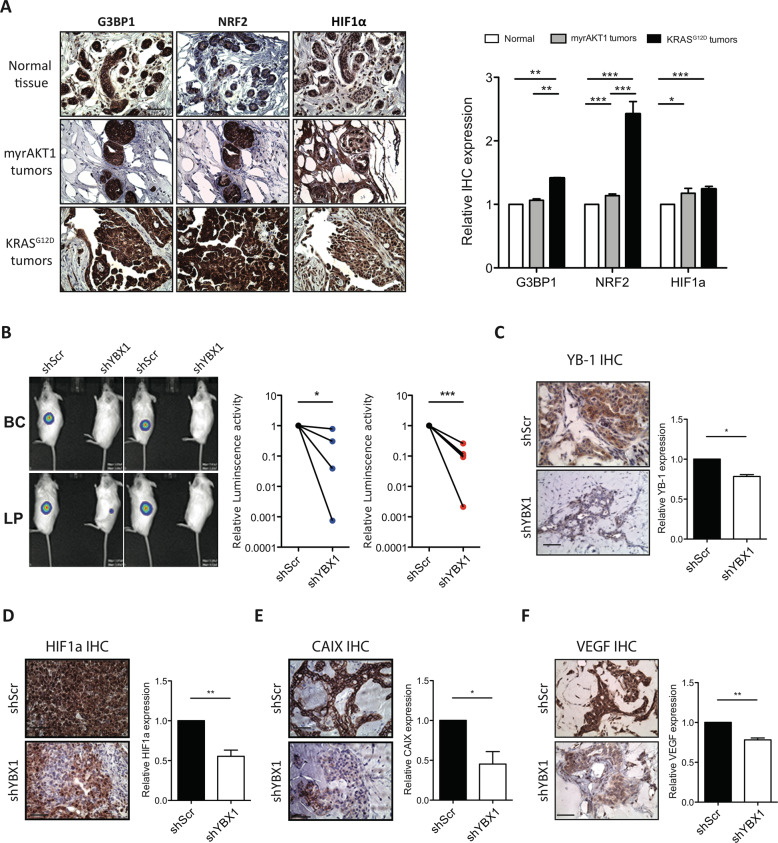
Increased YB-1 is necessary for *KRAS*^*G12D*^-induced human mammary tumorigenesis. **A** Representative views of G3BP1, NRF2 and HIF1α immunostaining of normal human mammary tissue (top) and 8-week tumours derived from *myrAKT1-* (middle) and *KRAS*^*G12D*^- (bottom) transduced normal mammary cells. Scale bar, 50 μm. The bar graph shows a quantification of G3BP1, NRF2 and HIF1α expression. Data are from individual tumours (Normal, *n* = 8; myrAKT1 tumours, *n* = 8; KRAS^G12D^ tumours, *n* = 8). **B** Representative photos of the bioluminescence signals obtained in mice injected SQ with *KRAS*^*G12D*^ + *shYB-1*- or *KRAS*^*G12D*^ + *shScr*-transduced primary human mammary cells 2 weeks earlier. The dot plot shows the bioluminescence activity of tumours derived from BCs (blue) and LPs (red). *N* = 4. **C**–**F** Representative images of YB-1 (**C**)-, HIF1α (**D**)-, CAIX (**E**)- and VEGF (**F**)-stained sections from different BC- or LP-derived tumours arising from *KRAS*^*G12D*^ + *shYB-1*- or *KRAS*^*G12D*^ + *shScr*-transduced cells. Scale bar, 100 μm. The bar graphs show quantification of YB-1 (**C**)-, HIF1α (**D**)-, CAIX (**E**)- and VEGF (**F**)-intensities in tumours derived from *KRAS*^*G12D*^ + *shYB-1*- or *KRAS*^*G12D*^ + *shScr* -transduced cells. *N* = 8. *P*-values in panels **A**–**E** were determined by Student’s *t* test, *P < 0.05, **P < 0.01, ***P < 0.001.

IHC analysis of de novo tumours derived from *KRAS*^*G12D*^-transduced human mammary cells showed increased levels of stress-related translational targets of YB-1, including HIF1α, NRF2, G3BP1 and membranous CAIX (Fig. 6A and Appendix Fig. S9A), compared to matching normal tissue. Modest increases in NRF2 and HIF1α expression were also seen in the *myrAKT1*-transduced human mammary cells (Fig. 6A), suggesting that the induction of a stress-related response can occur during the initiation of transformation towards both IDC or even DCIS.

To determine whether YB-1 is required for the de novo genesis of tumours derived directly from oncogenically perturbed normal human mammary cells, we used the same YB-1 knockdown strategy applied above to the MDA-MB-231 cells. Accordingly, purified BCs and LPs were transduced first with *shYB-1* or control (*shScr*) lentiviral vectors (Appendix Fig. S9B), and then, after 2 days in vitro, the cells were transduced at high efficiency with vectors encoding both *KRAS*^*G12D*^-mCherry and the Luc-YFP vector prior to being transplanted SQ into immunodeficient mice (2 × 10^4^−2.5 × 10^5^ cells/transplant). Two weeks following transplantation, cells derived from either BCs or LPs that had been co-transduced with the *shYB-1* vectors produced bioluminescent signals that were 2–1000-fold lower than those obtained from their counterparts transduced with *KRAS*^*G12D*^ and the control sh vector (Fig. 6B). H&E-staining showed that the cellularity of the BC and LP-derived tumours was greatly reduced compared to that of parallel tumours generated from the control cells (*KRAS*^*G12D*^ plus the *shScr* construct, Appendix Fig. S9C). The very small size of the tumours generated from the *KRAS*^*G12D*^ + *shYB-1-*co-transduced cells severely limited further mechanistic studies. However, a critical role for YB-1 in orchestrating a cytoprotective state via the HIF1α pathway was suggested by the reduced number of YB-1^+^ cells present in the test cell-derived harvests (Fig. 6C), along with corresponding diminished levels of HIF1α (Fig. 6D), CAIX (Fig. 6E), VEGF (Fig. 6F) and CD34 (the latter as a marker of blood vessels; Appendix Fig. S9D).

Together, these experiments establish that the initial tumorigenic activity displayed by xenografts of normal human mammary cells forced to express *KRAS*^*G12D*^ is highly dependent on an upregulation of YB-1 and this is associated with a YB-1-dependent expression of proteins that mediate the cellular stress response.

## Discussion

Here we show that increased expression of *YB-1* characteristic of poor prognosis breast cancers is associated with elevated KRAS or AKT activity and an activated stress response programme. In addition, we show that these associations drawn from patient data are reproduced in several experimental models of human breast cancer growing in vivo in transplanted immunodeficient mice. The latter include the established MDA-MB-231 human breast cancer cell line that has a *KRAS*^*G13D*^ mutation [16] and highly penetrant metastatic as well as tumorigenic activity in transplanted immunodeficient mouse hosts. In addition, we show that upregulation of YB-1 protein is a rapidly acquired feature of tumours produced in mice transplanted with T47D cells or primary normal human mammary cells forced to overexpress *KRAS*^*G12D*^. We also describe an entirely new model of de novo transformed normal human mammary cells that displays features of DCIS when either BCs or LPs are forced to express intrinsically deregulated AKT activity that also increases expression of YB-1 in their immediate progeny. Taken together, these findings indicate a role for YB-1 both at the point of initial transformation of normal human mammary cells, and one that is also sustained in more advanced types of breast cancer cells that have acquired metastatic potential.

Previous experiments in which YB-1 was overexpressed in the immortalised but non-tumorigenic MCF10A cell line transformed with HRAS showed that high YB-1 expression contributes to the disruption of the normal mammary cell architecture they produce and promotes an epithelial mesenchymal transition [9]. However, published patient breast cancer datasets [20, 21, 43], show a clear link between increased *YB-1* expression and amplified *KRAS*, but no association with *HRAS* or *NRAS*. In the present study, we provide evidence of a similar loss of a normal acinar architecture by *KRAS*^*G12D*^-transduced human mammary cells both in 3D cultures in vitro and following transplantation into mice. Interestingly, we find this is accompanied by a corresponding increase in expression of *YB-1* in vivo and its associated activated stress response targets. In addition, we show upregulated YB-1 is also an obligate requirement for the creation of a DCIS outcome from in vivo transplanted human mammary cells forced to express a constitutively activated form of AKT. These findings are further supported by similar results obtained by examining 3D cultures and tumours generated in vivo from T47D cells transduced with *KRAS*^*G12D*^. These findings thus add new weight to a growing body of evidence that induced expression of YB-1 confers a cytoprotective mechanism to transformed cells exposed to increased stresses, such as those created during tumour growth or metastasis, or growth under adverse conditions in vitro.

Specifically documented in many of these models were YB-1-mediated increases in levels of G3BP1, NRF2, HIF1α and CAIX RNA and/or protein. Conversely, suppression of YB-1 impeded the enhanced expression of HIF1α and CAIX in tumours generated from both *de novo* transformed primary cells as well as established breast cancer cell lines. Thus, the finding of heightened expression of HIF1α and HIF target genes in patients’ breast cancers with amplified KRAS can now be directly related to a perturbation of KRAS activity and likely that of more commonly mutated downstream elements in the KRAS pathway. Together, these findings also support a more generalised model in which activated forms of KRAS or AKT directly and/or indirectly increase levels of YB-1 levels, which, in turn, promote an elevated HIF1α response and potentially other cytoprotective programmes required for tumour formation in vivo. Importantly, the shared high expression of *HIF1α* and its target genes in triple-negative breast cancers [44] underscores the importance of our findings and the opportunities afforded by the models we describe here for the future pursuit of additional mechanistic and translational investigations. In this context, it is interesting to note that we have recently found that HIF1α induction in human sarcoma cells is mediated by direct binding of YB-1 to the 5′-UTR of *HIF1A* transcripts to enhance their translation [5]. In addition, we have also demonstrated that YB-1 regulates stress granule formation and tumour progression in sarcomas by translationally activating G3BP1 and SG formation [8]. KRAS has also been shown to promote SG formation following exposure of colon and pancreatic cancer cells to stress-inducing stimuli [7]. YB-1 might thus represent a common intermediate in the activation of many cytoprotective programmes required for multiple types of tumour cells to survive and grow when they are exposed to different stress conditions in vivo.

Taken together, these findings add strong weight to the concept that YB-1 represents a relevant target for therapeutic intervention in breast cancer. We recently reported that class I histone deacetylase (HDAC) inhibitors induce hyperacetylation within the RNA binding CSD of YB-1 in sarcoma cells [6], thus reducing its binding to cytoprotective mRNAs and downstream effects of their translational activation. In addition, the present findings indicate that targeting HIF1α or its downstream effectors may represent alternative clinical targets in breast cancer, and further highlight the possibility that these approaches may also be effective on cells at very initial stages of breast cancer formation.

## Supplementary information

Supplementary figures legends

Supplementary figures
