# Supplementary material for: De novo and cell line models of human mammary cell transformation reveal an essential role for Yb-1 in multiple stages of human breast cancer
Source: Cell Death Differ. 2021 Jul 22;29(1):54–64. doi: 10.1038/s41418-021-00836-6 (PMC8738742; doi:10.1038/s41418-021-00836-6)
Supplement: Supplementary file 1 — Supplementary figures legends [file 41418_2021_836_MOESM1_ESM.docx]

**De novo and cell line models of human mammary cell transformation reveal an essential role of YB-1 in multiple stages of human breast cancer**

**Supplementary Figure Legends**

**Appendix Figure S1. High levels of *YBX1* transcripts are associated with metastatic human breast cancers and *KRAS-*amplified tumors.**

**A-B**. *YBX1* mRNA expression according to the ER (**B**) or metastatic status (**C**) of invasive breast carcinomas in the TCGA dataset.

**C.** *YBX1* mRNA expression according to gene copy numbers of *TP53, ERBB2*, *PIK3CA* and *AKT1* in invasive breast carcinomas in the TCGA dataset. All *YBX1* values are shown as RPKMs.

**D.** Representative table of *YBX1* alteration co-occurrence in metastatic breast cancer.

**Appendix Figure S2. *KRAS* and AKT1 amplifications are associated with a YB-1-regulated stress response.**

**A.** *VEGFA* (left panel) and *NFE2L2* (right panel) mRNA expression according to *KRAS* copy number status in invasive breast carcinomas in TCGA dataset. Values for *VEGFA, and NFE2L2* are shown as RPKMs.

**B.** *CAIX* (left panel), and *VEGFA* (right panel) transcript levels (RPKM values) shown according to *KRAS* copy number status in the invasive breast carcinoma data set in METABRIC.

**C.** Scatter plot of *YBX1* and *CAIX* mRNA expression in amplified-*KRAS* invasive breast carcinomas (METABRIC dataset).

**D.** *HIF1A* (left panel), *CAIX* (middle panel), and *VEGFA* (right panel) transcript levels are shown according to *YBX1* copy number status in the TCGA invasive breast carcinoma dataset. All transcript values are shown as RPKMs.

**E.** *VEGFA* (left panel) and *CAIX* (right panel) transcript level (RPKM values) shown according to *AKT1* copy number status in the invasive breast carcinoma data set in METABRIC.

**F.** *YBX1*, *HIF1A*, *CAIX* and *VEGFA* transcript levels are shown according to *AKT1* copy number status in the TCGA invasive breast carcinoma dataset. All transcript values are shown as RPKMs.

**G-H**. Scatter plot of *YBX1* and *CAIX* mRNA expression in amplified-*AKT1* invasive breast carcinomas in METABRIC (**G**) or (**H**) TCGA datasets.

**Appendix Figure S3. YB-1 is required for dissemination of intravenously injected MDA-MB-231 cells.**

**A**. YB-1 expression analyzed by Western blot assessment of extracts of sh*YBX1*- or sh*Scr*-transduced MDA-MB-231 cell harvests.

**B**. YB-1 expression analyzed by FACS analysis of individual sh*YBX1*– or sh*Scr-*transduced MDA-MB-231 cell harvests.

**C**. Representative pictures of bioluminescence signals in mice injected subcutaneously with MDA-MB-231 cells transduced with sh*Scr* or sh*YBX1*. Dot plot shows the measured bioluminescence in these tumours 26 days post-transplant. N=6.

**D**. Weights of the tumours shown in (**C**). N=14.

**E.** Representative views of YB-1 levels in the IHC-stained *in vivo* progeny of sh*YBX1*- or sh*Scr-*transduced MDA-MB-231 cells. Bar graph shows quantification of YB-1 expression.

**F.** Representative pictures 45 days post-transplant of bioluminescence signals measured in mice injected intravenously with sh*YBX1*- or sh*Scr-*transduced MDA-MB-231 cells. Dot plot showing levels of bioluminescence of tumors derived from MDA-MB-231 cells transduced with sh*Scr* or a sh*YBX1* and assessed 47 days post-transplant. N=3.

**G.** Representative H&E-stained photomicrographs of lungs of mice injected intravenously with sh*YBX1*- or sh*Scr*-transduced MDA-MB-231 cells.

**H.** Comparison of YB-1 levels (staining intensity) in lung tumors shown in **G**.

**Appendix Figure S4. Inhibition of *YBX1* expression downregulates expression of *PPIF*, *SLC3A2* and *SLC7A1*.**

**A**. Hierarchical clustering of proteomics data obtained on tumour cells generated from sh*YBX1- or* sh*Scr*-transduced MDA-MB-231 cells.

**B**. Correlation plot between transcripts and proteins identified by RNAseq and proteomic analysis of tumours derived from sh*YBX1*- or sh*Scr*-transduced MDA-MB-231 cells.

**C-D.** Genes whose transcript and protein expression in MDA-MB-231 cells after transduction with sh*YBX1* was either upregulated (**C**) or down-regulated (**D**) by comparison to sh*Scr*-transduced (control) cells from analyses of matched pairs of RNAseq and proteomics data.

**E.** *SLC3A2,* *PPIF* and *SLC7A1* mRNA expression according to *YBX1* copy number status in invasive breast carcinomas in the TCGA data. Values for *SLC3A2* and *PPIF* are shown as RPKMs.

**Appendix Figure S5**. ***KRAS^G12D^*-overexpression lead to YB-1 increase *in vitro* only in stressed conditions.**

**A** Western blots of GFP-control and *KRAS^G12D^*-transduced T47D cells, showing YB-1, P-ERK1/2, ERK1/2 and RAS (relative to Actin). Dot plots showing P-ERK (relative to Actin) and YB-1 (relative to Actin) levels. N=7.

**B** Western blots showing HIF1α, YB-1 and RAS levels (relative to GAPDH) of GFP-control and *KRAS^G12D^*-transduced T47D cells grown in ultra-low attachment plates for 48h. N=5.

**Appendix Figure S6**. ***KRAS^G12D^*-transduced primary human mammary cells display high levels of YB-1 only *in vivo*.**

**A.** Western blots showing YB-1 and RAS levels (relative to H3) in control and *KRAS^G12D^*-transduced human BCs and LPs from 3 normal donors, as assessed 3 days post-transduction.

**B** Representative immunofluorescence images of control and *KRAS^G12D^*-transduced BCs (top) and LPs (bottom) assessed 15 days post-transduction, and cultured in 3D in Matrigel. Staining was performed using an anti-YB-1 antibody, Phalloidin (F-ACTIN) and DAPI (DNA).

**C**. Representative images of YB-1 immunostaining of 2 week-old xenografts of *KRAS^G12D^*-transduced primary human mammary cells. Scale bar, 200 μm (left) or 100 μm (right).

**D.** Design of the lentiviral pINDUCER21-KRAS-2A-KO2 reporter construct.

**E.** Representative FACS profile of human mammary MCF10A cells stably expressing an inducible KRAS-2A-KO2 construct after being maintained in the presence or absence of doxycycline.

**F.** Western blots showing RAS levels (relative to ACTIN) in the same cells as in (**E**).

**Appendix Figure S7. Transplants of primary human mammary cells expressing *myrAKT1* produce DCIS-like structures.**

**A**. Representative photos of bioluminescence signals obtained in mice injected subcutaneously 5 or 7 weeks earlier with Luc-YFP- and *myrAKT1*-transduced human mammary cells. Graph plot shows bioluminescence activity from tumours derived from BCs (blue) and LPs (red). N = 5 donors.

**B-C**. Representative IHC images of sections stained for SMA, CK14, CK8-18 and p63 (**B**); or ER, PR, and Ki67 (**C**) in tumours derived from transplants of either BCs or LPs-transduced with *myrAKT1*. Scale bar, 100 μm.

**Appendix Figure S8. YB-1 induction model.**

**A.** Representative FACS profile (left panel) and Western blots (right panel) of human mammary MCF10A cells expressing inducible YBX1-2A-KO, cultured with or without doxycycline.

**B**. Representative IHC images of sections stained for YB-1 in tumors generated from LPs (left) or BCs (right) transduced with *myrAKT1* vector an inducible YBX1, and transplanted into mice given water with (bottom) or without doxycycline (top).

**Appendix Figure S9. Suppression of YB-1 expression impairs *in vivo* tumour formation by *KRAS^G12D^*-transduced normal human mammary cells.**

**A.** Representative views of CAIX immunostaining of normal human mammary tissue (left) and 8-week tumours derived from *myrAKT1-* (middle) *KRAS^G12D^*- (bottom) transduced normal mammary cells. Scale bar, 50 μm.

**B.** Western blot showing YB-1 expression in cells expanded *in vitro* from isolated BCs and LPs transduced with sh*YBX1 or* sh*Scr* using cells from 3 normal donors.

**C.** Representative images of H&E-stained sections from different BC- or LP-derived tumours arising from *KRAS^G12D^*+sh*YBX1-* or *KRAS^G12D^*+sh*Scr*-transduced cells. Scale bar, 200 μm.

**D.** Representative images of CD34-stained sections from different BC- or LP-derived tumours arising from *KRAS^G12D^*+sh*YBX1*- or *KRAS^G12D^*+sh*Scr*-transduced cells. Scale bar, 200 μm. Bar graphs shows quantification of CD34 intensities in tumours derived from *KRAS^G12D^*+sh*YBX1-* or *KRAS^G12D^*+sh*Scr*-transduced cells from 6 normal donors.
