# Supplementary material for: De novo and cell line models of human mammary cell transformation reveal an essential role for Yb-1 in multiple stages of human breast cancer
Source: Cell Death Differ. 2021 Jul 22;29(1):54–64. doi: 10.1038/s41418-021-00836-6 (PMC8738742; doi:10.1038/s41418-021-00836-6)

Appendix Figure S1

A

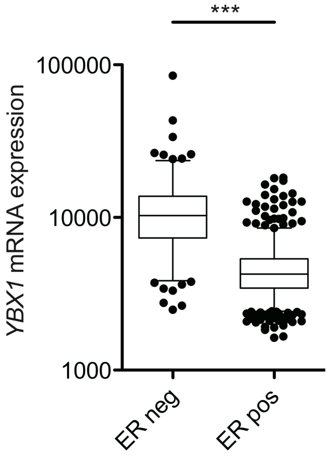

B

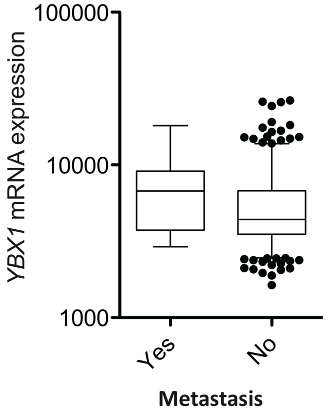

C

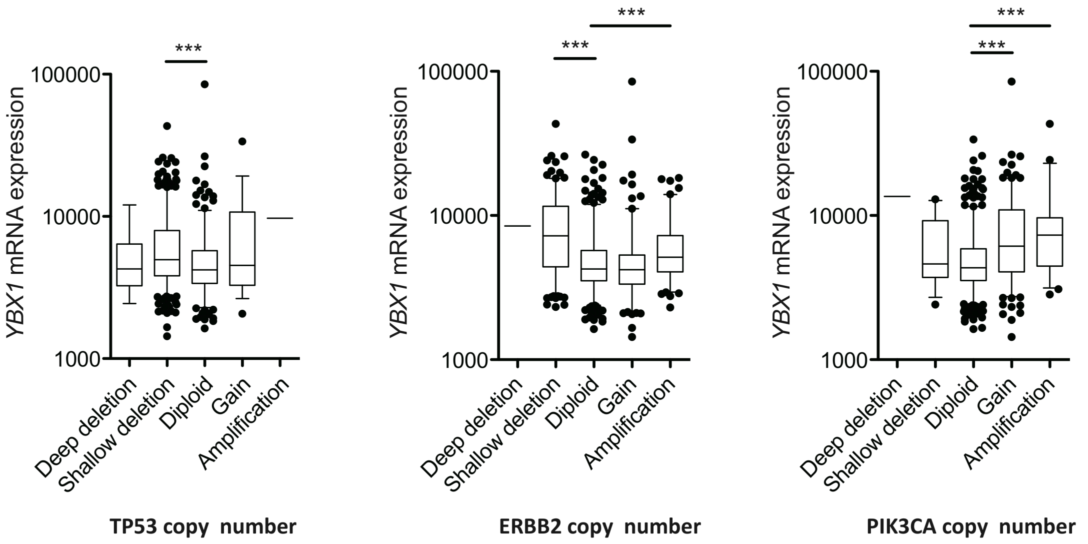

D

YBX1 co-occurrent alterations

Gene	p-value	Log odds ratio
KRAS	0,026	2.71
TP53	0,150	1.43
PIK3CA	0,609	-0.37
ERBB2	0,369	1.04

Metastatic Breast Cancer

Appendix Figure S2

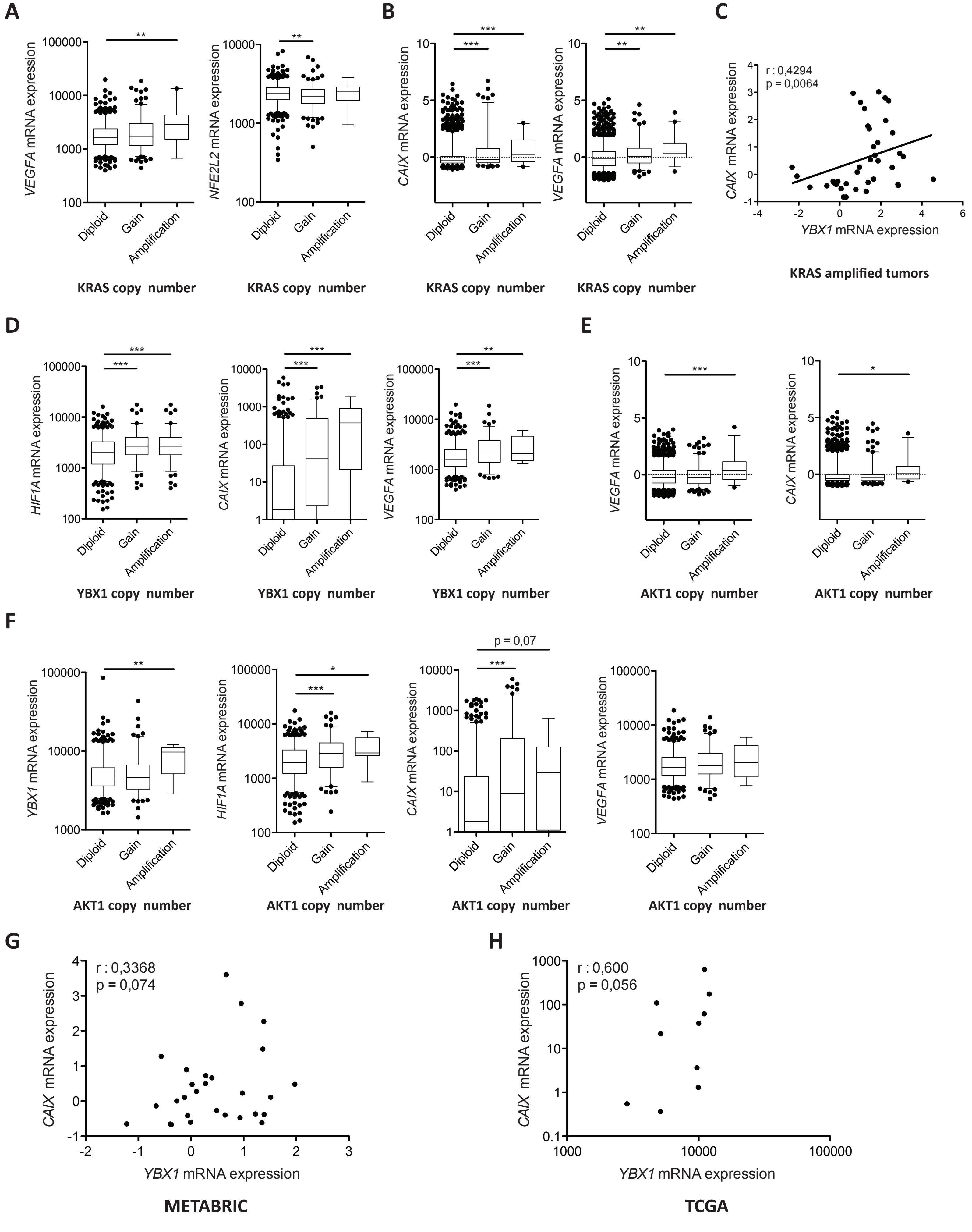

Appendix Figure S3

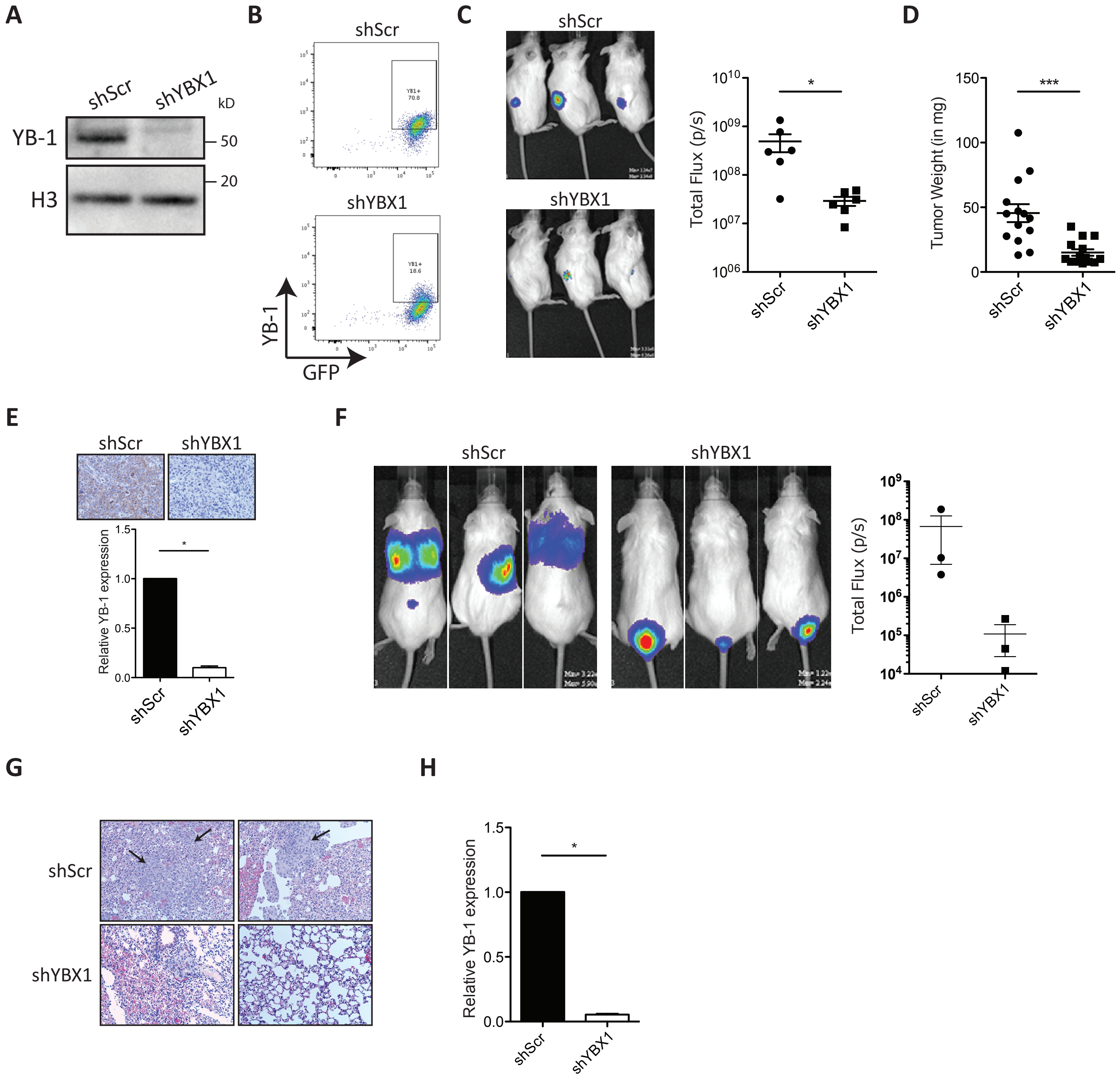

Appendix Figure S4

A

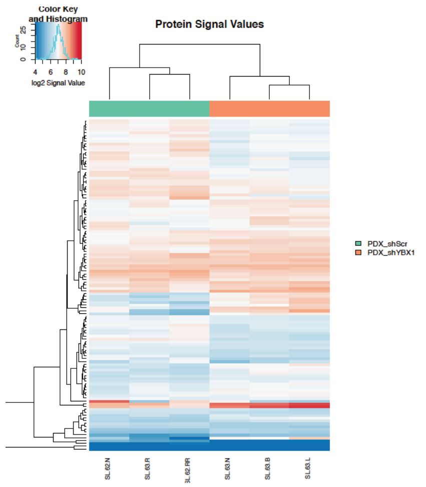

B

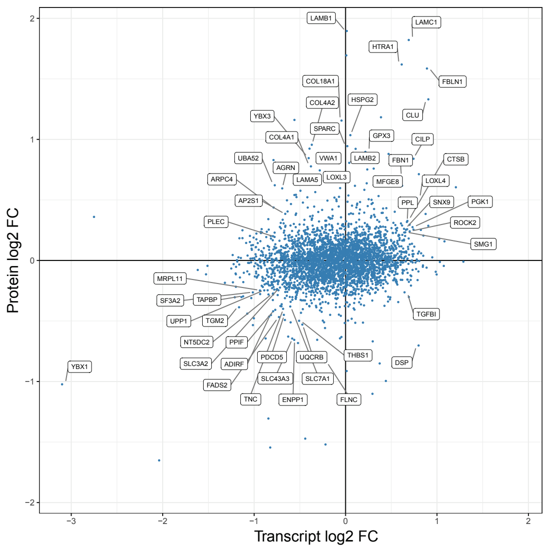

C

	logFC_tumor	logFC_cell	logFC_protein	name
CLU	0,686204948	0,561829289	1,13	clusterin
AQP1	0,685713585	0,030616296	-1,19	aquaporin 1 (Colton blood group)
FBN1	0,582909057	0,316026985	0,33	fibrillin 1
HTRA1	0,566998828	0,45267254	1,41	HtrA serine peptidase 1
AKR1C3	0,506919019	0,282867522	0,53	aldo-keto reductase family 1 member C3
CTSB	0,465610178	0,468705607	0,34	cathepsin B
KRT7	0,428710908	0,251378605	0,39	keratin 7
PPL	0,420717705	-0,096063579	0,31	periplakin
PTER	0,395473707	0,477052255	0,35	phosphotriesterase related
FLNB	0,372621271	-0,025828427	0,27	filamin B
EML4	0,366910152	0,095128757	0,45	echinoderm microtubule associated protein like 4

D

	logFC_tumor	logFC_cell	logFC_protein	name
SLC7A1	-0,386736896	0,021588221	-0,36	solute carrier family 7 member 1
BDH1	-0,39381943	0,167863328	-0,31	3-hydroxybutyrate dehydrogenase 1
KRI1	-0,397984911	-0,227047716	-0,32	KRI1 homolog
SLC3A2	-0,398601902	-0,081431865	-0,29	solute carrier family 3 member 2
TAPBP	-0,414419639	-0,025529158	-0,28	TAP binding protein
DNAH2	-0,461453218	-0,107131451	1,19	dynein axonemal heavy chain 2
PPIF	-0,477266682	-0,024606039	-0,39	peptidylprolyl isomerase F
BRI3BP	-0,56225646	0,371085294	-0,59	BRI3 binding protein
YBX1	-2,034849551	-0,223340873	-0,99	Y-box binding protein 1

E

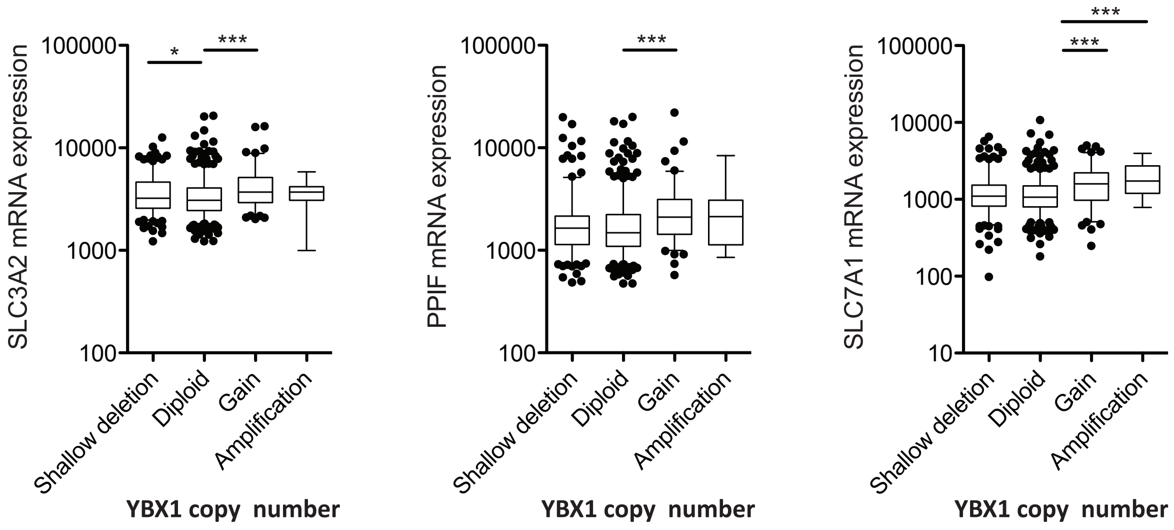

Appendix Figure S5

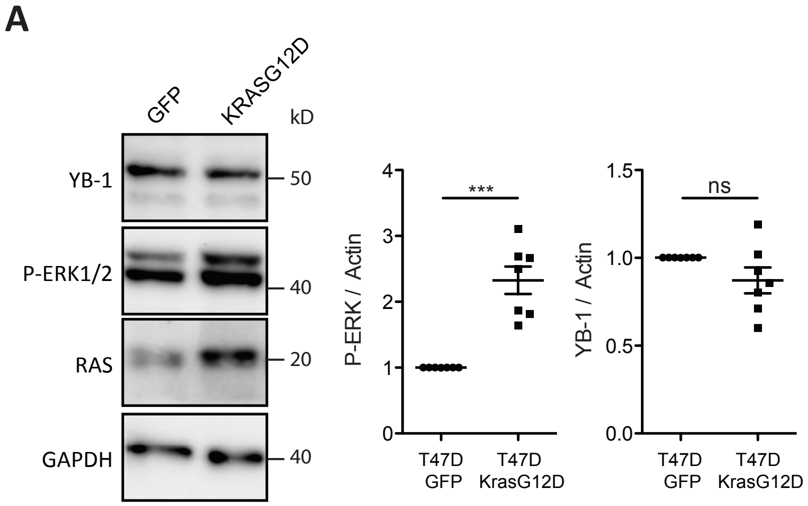

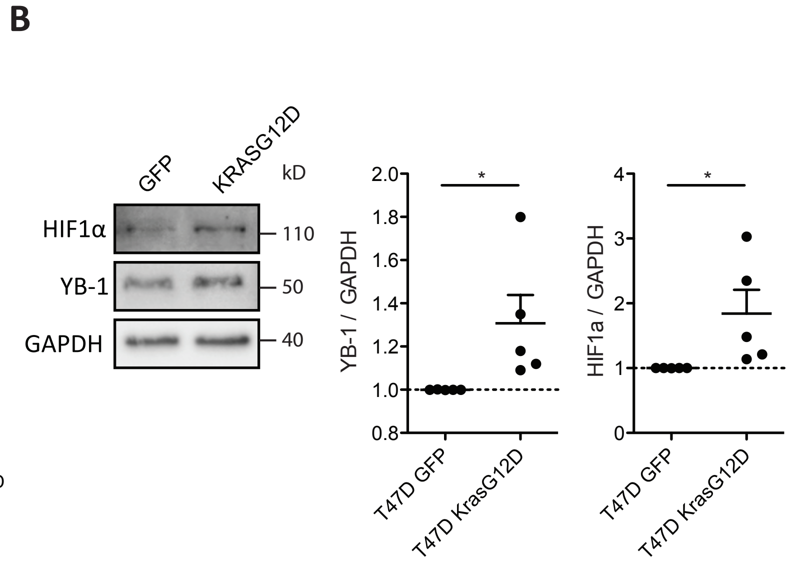

Appendix Figure S6

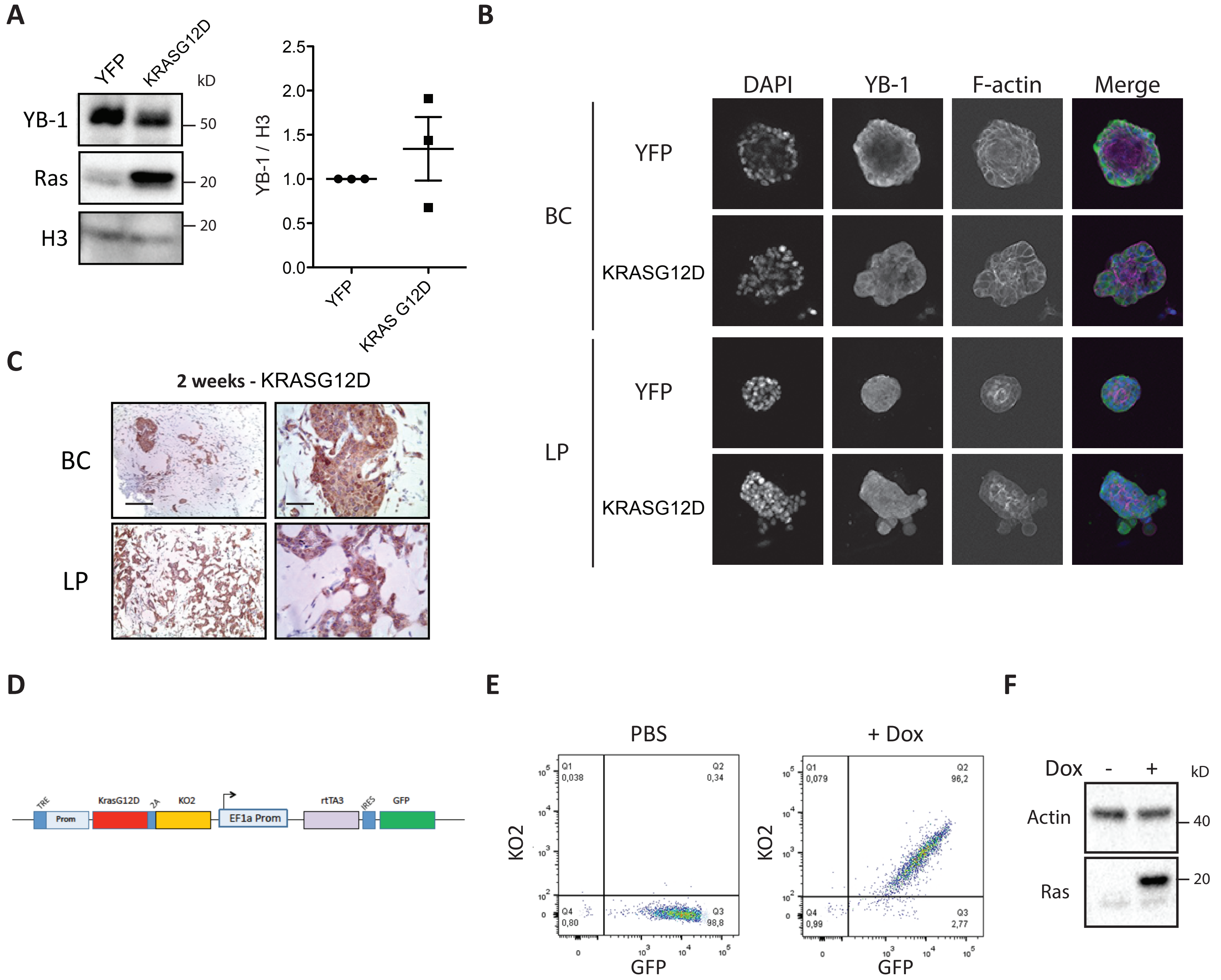

Appendix Figure S7

A

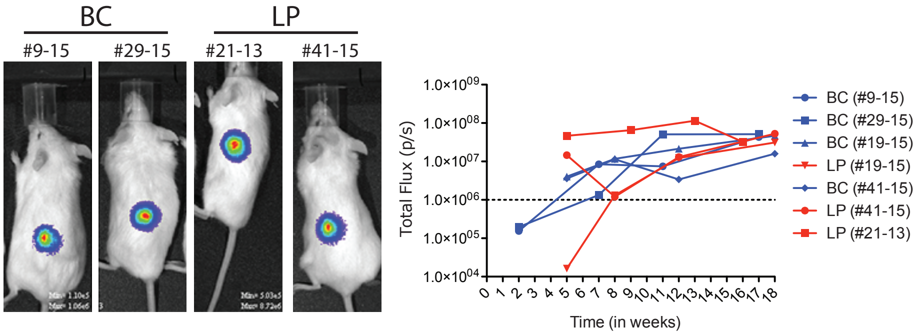

B

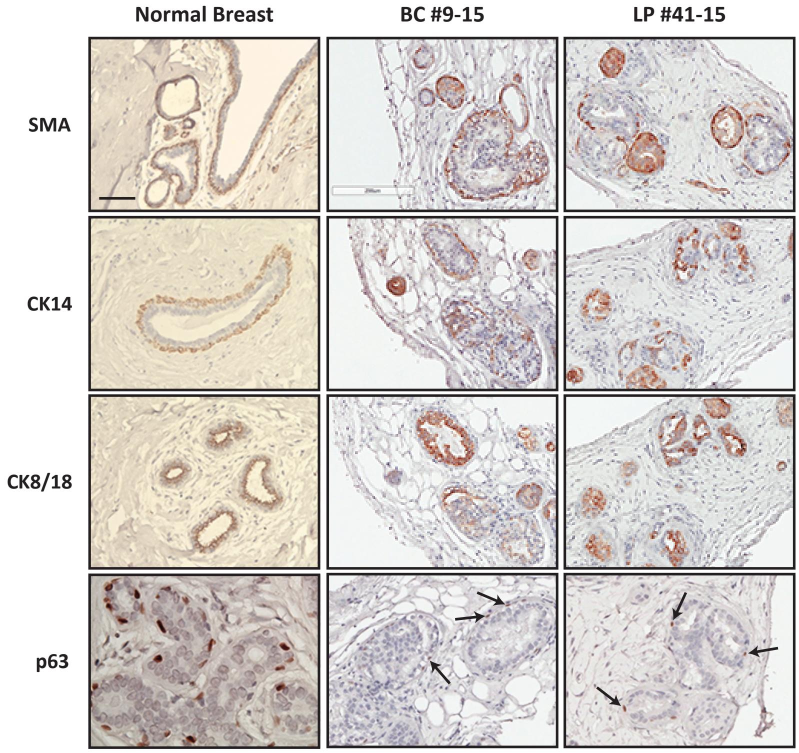

C

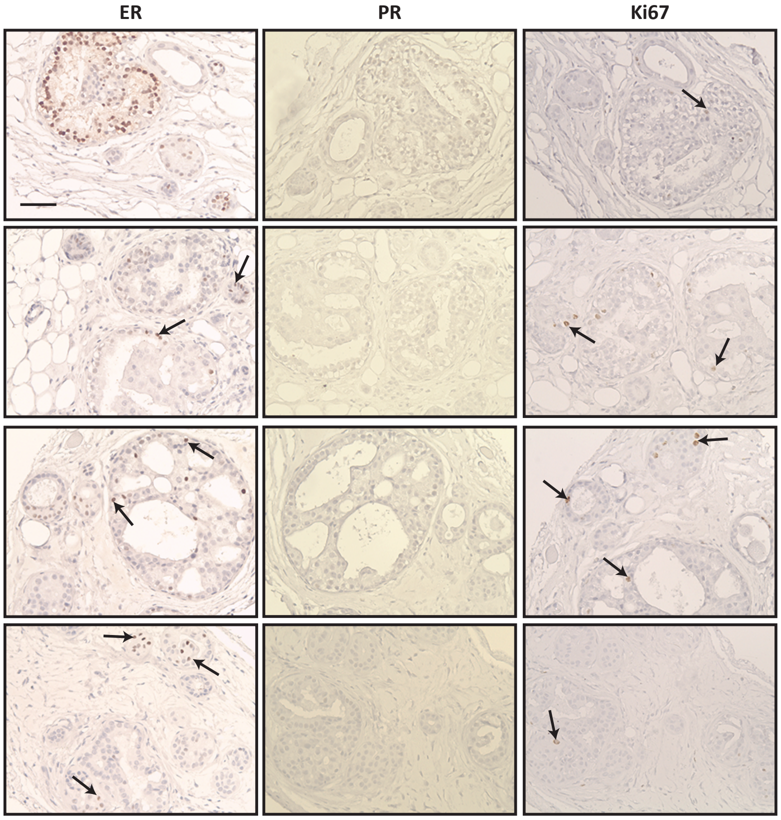

Appendix Figure S8

A

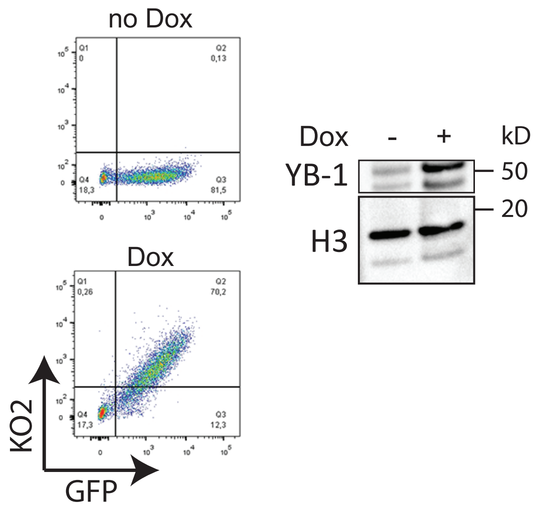

B

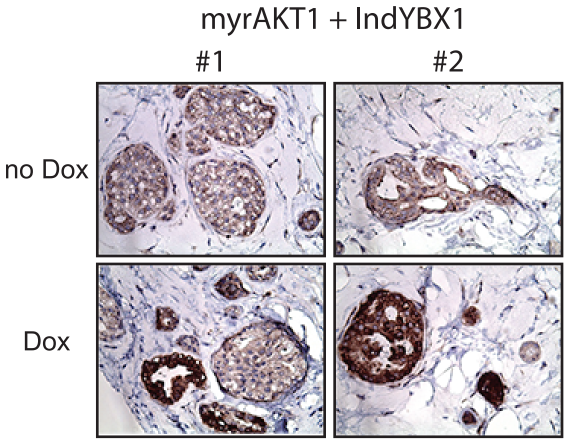

Supplementary Figure S9

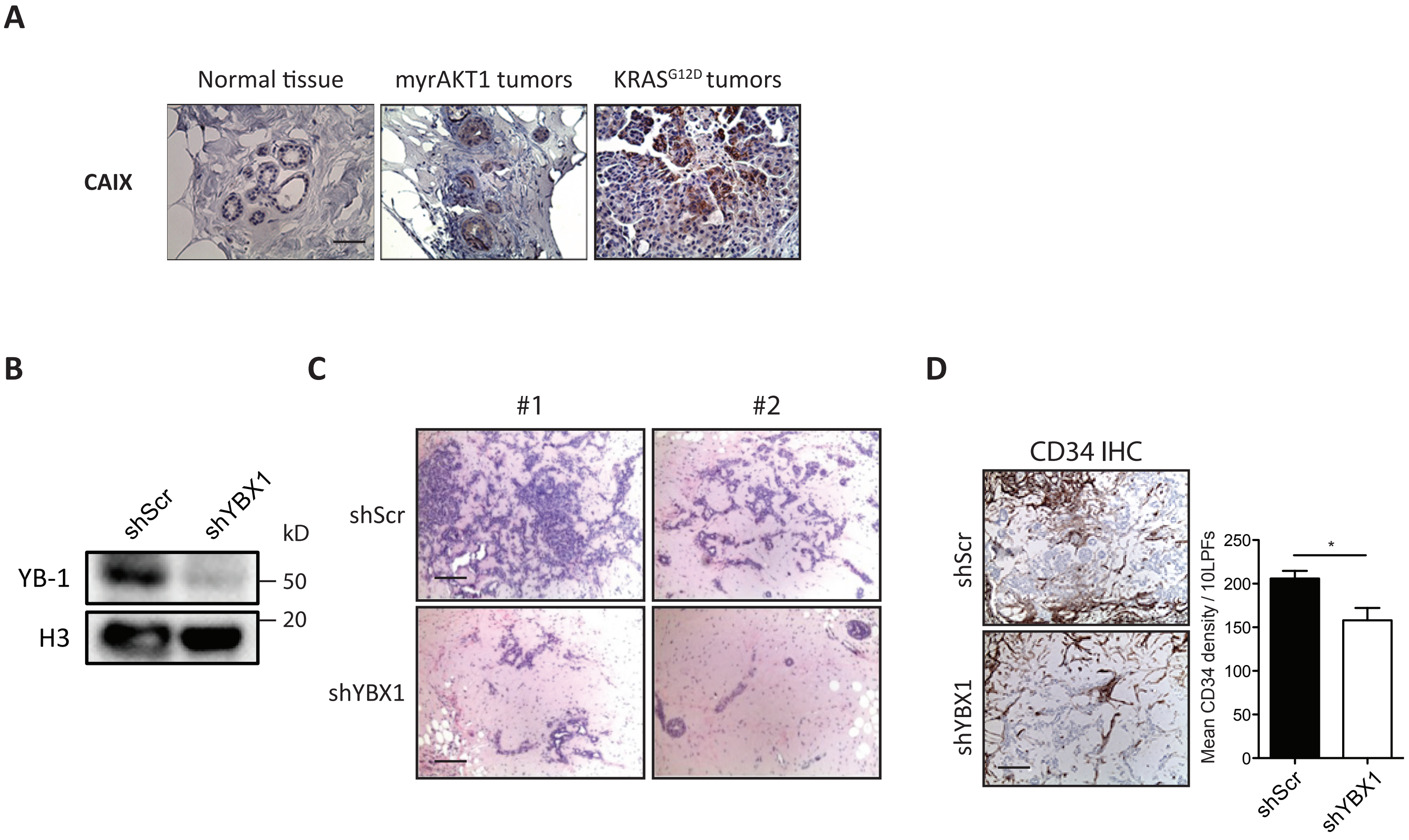

Supplement: Supplementary file 2 — Supplementary figures [file 41418_2021_836_MOESM2_ESM.pdf]
